# Impact of a National Stepped‐Care Model of Opioid Use Disorder Treatment on Access to Medication Therapies Among Homeless‐Experienced Veterans

**DOI:** 10.1111/1475-6773.70171

**Published:** 2026-10-13

**Authors:** Haojia Li, Jacob D. Baylis, Sophia Huebler, Adam J. Gordon, Eric Hawkins, Hildi J. Hagedorn, Stefan G. Kertesz, Ying Suo, Yue Zhang, Audrey L. Jones

**Affiliations:** ^1^ Department of Internal Medicine University of Utah School of Medicine Salt Lake City Utah USA; ^2^ Department of Population Health Sciences University of Utah School of Medicine Salt Lake City Utah USA; ^3^ Informatics, Decision Enhancement, and Analytic Sciences (IDEAS) Center VA Salt Lake City Health Care System Salt Lake City Utah USA; ^4^ Program for Addiction Research, Clinical Care, Knowledge and Advocacy (PARCKA), Division of Epidemiology, Department of Internal Medicine University of Utah Salt Lake City Utah USA; ^5^ Health Systems Research (HSR) Seattle Center of Innovation for Veteran‐Centered and Value‐Driven Care Veterans Affairs (VA) Puget Sound Health Care System Seattle Washington USA; ^6^ Center of Excellence in Substance Addiction Treatment and Education VA Puget Sound Health Care System Seattle Washington USA; ^7^ Department of Psychiatry and Behavioral Sciences University of Washington School of Medicine Seattle Washington USA; ^8^ Center for Care Delivery & Outcomes Research Minneapolis VA Health Care System Minneapolis Minnesota USA; ^9^ Birmingham VA Health Care System Birmingham Alabama USA; ^10^ Department of Medicine University of Alabama at Birmingham Heersink School of Medicine Birmingham Alabama USA; ^11^ Department of Health Behavior University of Alabama at Birmingham School of Public Health Birmingham Alabama USA

**Keywords:** buprenorphine, homeless, implementation science, opioid use disorder

## Abstract

**Objective:**

To determine whether the Veterans Health Administration's (VHA) Stepped Care for Opioid Use Disorder Train‐the‐Trainer (SCOUTT) initiative increased medication treatment for opioid use disorder (MOUD) among persons experiencing homelessness (PEH).

**Study Setting and Design:**

We conducted a national retrospective cohort study of MOUD, including buprenorphine, naltrexone, or methadone, in federal fiscal years (FY) 2016–2020. In late FY2018, VHA regional directors nominated facilities to receive SCOUTT implementation support to expand prescribing of MOUD (primarily buprenorphine) in select primary care, pain, and general mental health settings. We used mixed‐effects quasi‐Poisson regression models with entropy balancing to estimate the Average Treatment Effect on the Treated in the years after SCOUTT was implemented (FY2019–2020), controlling for pre‐implementation levels of MOUD. Secondary analyses applied synthetic difference‐in‐differences (DiD) to estimate changes associated with SCOUTT.

**Data Sources and Analytic Sample:**

We used yearly pooled cross‐sectional data from VHA electronic health records, FY2016–2020. The sample included annual cohorts of PEH with an opioid use disorder diagnosis and ≥ 1 primary care visit in the pre‐implementation (FY2016–2018, *n* = 31,354) or post‐implementation (FY2019–2020, *n* = 20,746) periods.

**Principal Findings:**

From FY2016–2020, MOUD receipt increased more in SCOUTT facilities (*n* = 30, 45.0% to 61.1%) than in non‐SCOUTT facilities (*n* = 811, 41.9% to 49.7%). In post‐implementation years, PEH in SCOUTT facilities, compared to non‐SCOUTT facilities, were more likely to receive any MOUD (risk difference [RD] = 6.16, 95% CI = 2.33–9.99), including buprenorphine (RD = 7.16, 95% CI = 4.35–9.97). There were no significant differences in methadone (RD = −0.19, 95% CI = −1.03–0.65) or naltrexone (RD = 0.10, 95% CI = −1.85–2.05). Synthetic DiD offered similar results: any MOUD DiD = 7.85, 95% CI = 2.15–13.55; buprenorphine DiD = 9.31, 95% CI = 3.56–15.06.

**Conclusions:**

A national initiative to advance a stepped‐care model that encourages MOUD initiation outside specialty addiction settings improved access to buprenorphine among PEH. Similar initiatives could help to close treatment gaps in health care settings serving PEH.

**Callout Box**

- What is known on this topic.
  ○ Opioid use disorder (OUD) continues to drive preventable morbidity and mortality in the United States, with persons experiencing homelessness (PEH) disproportionately affected.
  ○ A Veterans Health Administration initiative to implement a stepped‐care model of OUD, which encourages initiation of medication therapies (MOUD) in low‐intensity settings, improved treatment access and retention among general outpatients.
  ○ It is not yet known whether large‐scale implementation of stepped‐care approaches for OUD is effective for increasing MOUD access among PEH, specifically.
- What this study found
  ○ VHA facilities that received stepped‐care training and implementation support saw greater increases in buprenorphine treatment for OUD, compared to other VHA facilities.
  ○ Study results were consistent when applying two quasi‐experimental design methods to account for site differences in case‐mix, infrastructure, and pre‐intervention treatment patterns.
  ○ Implementation strategies to expand MOUD in low‐intensity settings, such as primary care and mental health clinics, could be effective for PEH for whom specialized addiction treatment settings are less desirable.

## 1 Introduction

The opioid crisis is among the greatest public health challenges in the United States due to substantial personal and societal harms, and gaps in access to evidence‐based treatments for opioid use disorder (OUD) among high‐need populations [1]. Opioids accounted for 78% of U.S. deaths attributed to drug use, and more than half the global death toll from drug use disorders in 2021 [2]. More than five million U.S. adults met OUD criteria adopted by the National Survey on Drug Use and Health [3]. Persons with ongoing or recent experience of homelessness (PEH) have been disproportionately impacted by the opioid crisis. They are at higher risk for OUD and opioid‐related harms [4] including overdose [5, 6] and death [7]. Medication therapies for OUD (MOUD), such as buprenorphine, naltrexone, and methadone, help to reduce opioid use and overdose risk [8], but uptake is low [3]. PEH are less likely than housed populations to receive MOUD [9, 10].

Barriers to MOUD among PEH include transportation and logistical challenges, getting to clinics that dispense methadone, competing priorities, stigma, discrimination in addiction treatment settings, and strict program requirements (e.g., frequent visits, urine toxicology, therapy) that impact patients' engagement in treatment and ability to work [11, 12, 13, 14]. However, when MOUD is provided in office‐based, mobile, and shelter settings accessible to PEH, MOUD engagement, retention and mortality outcomes can improve [15, 16]. Thus, low‐barrier models of MOUD care, such as those provided outside traditional addiction treatment settings, could help to expand MOUD reach and retention and improve outcomes for PEH [6, 15].

The Veterans Health Administration (VHA) is the largest integrated health care system in the U.S., annually providing health and social services to nearly 300,000 military Veterans with homeless experience and housing instability [17]. Recognizing suboptimal utilization of MOUD across VHA [18], some VHA facilities joined a program to support clinical teams offering a stepped‐care model of OUD treatment in office‐based settings. Stepped‐care models emphasize low‐intensity settings of care for patients with mild to moderate OUD, with pathways to specialty substance use disorder (SUD) treatment programs for patients with greater complexity and need for more intensive services [19]. The VHA's Stepped‐Care for Opioid Use Disorder Train the Trainer (SCOUTT) initiative employed implementation strategies to encourage prescribing of MOUD in primary care, pain and mental health clinics [19]. Prior evaluations found SCOUTT implementation (e.g., external implementation facilitation, education, mentoring) improved patients' access to buprenorphine MOUD [20], and contributed to a cultural shift in prescribing practices toward low‐intensity and nonspecialized settings of care [21]. It is not yet known whether large‐scale implementation of stepped‐care approaches for OUD is effective for increasing MOUD access among PEH, specifically. Outside the VHA, studies of office‐based provision of MOUD among PEH have been limited to a single program [15, 22, 23]. Within the VHA, evaluations of the stepped‐care model [20, 24, 25, 26] have not focused on PEH, but approaches that can increase their access to MOUD merit attention.

The goal of this study was to compare receipt of MOUD among PEH in VHA facilities in which a clinical team participated in the SCOUTT initiative to VHA facilities that did not participate. Because clinical teams volunteered to participate in the initiative, we sought to control for characteristics that could influence site participation using two approaches: (1) entropy balancing, which assigns weights so that PEH in SCOUTT and non‐SCOUTT facilities are exactly balanced on key characteristics; and (2) synthetic difference‐in‐differences methods which assign time weights in the pre‐implementation years so that SCOUTT and non‐SCOUTT facilities are balanced on pre‐implementation trends in MOUD. Evaluation findings from the VHA could inform community mental health centers, Health Care for the Homeless programs in Federally Qualified Health Centers and other health care systems on best practices for scaling up MOUD provision in office‐based settings serving PEH.

## 2 Methods

### 2.1 Study Design & Settings

We conducted a cohort study of MOUD among PEH in VA primary care using patient‐ and facility‐level data from the VHA's national electronic health records (EHR) from federal fiscal years (FY) 2016–2020. Data were part of a larger parent study of mental health and substance use disorder care in VA primary care environments [27]. Research activities were deemed exempt by the Institutional Review Board at the corresponding author's institution (The University of Utah IRB_00138059). This study followed the Strengthening the Reporting of Observational Studies in Epidemiology (STROBE) guidelines.

The VHA operates 1380 facilities across 18 service regions. In 2018, the 18 VHA regional directors each nominated a clinical team consisting of prescribing providers, nurse care managers, and pharmacy specialists to receive SCOUTT training. Teams were charged with implementing office‐based MOUD (primarily buprenorphine) in a primary care, pain, or mental health outpatient clinic setting in Phase 1, and expanding SCOUTT practices to these settings in other facilities in later phases [19]. The SCOUTT initiative supported implementation by offering education, assistance with workflow redesign, coaching, and external facilitation to problem‐solve solutions to local challenges [19, 25].

The present study included VHA facilities where a clinical team received SCOUTT training in Phase 1 (end of 2018) and, for comparison, facilities with no SCOUTT participation. We focused on FY 2016–2020 to ensure at least two full years of data pre‐ and post‐SCOUTT implementation.

### 2.2 Cohort

This study applied a pooled cross‐sectional data structure. PEH were included if they met the cohort criteria in at least one FY from 2016–2020. For a given FY, we identified VHA patients with evidence of homelessness or unstable housing, any diagnosis of OUD (F11.1x, F11.2x), and enrollment in primary care during the FY. Enrollment in primary care was defined as having an active primary care panel assignment and ≥ 1 primary care visit in the same year as evidence of unstable housing and the OUD diagnosis. These procedures were then repeated for the other FYs. The eligible records from all FYs were then combined (i.e., pooled) into a single analytic dataset.

Evidence of homeless experience and unstable housing was determined from International Classification of Diseases (ICD) codes in the EHR, receipt of VHA homeless services documented with clinic location codes, and responses to questions on VA's annual homeless screener (Table S1) [17]. Prior studies of the accuracy of EHR records (e.g., ICD codes, homeless service encounters) for capturing homelessness and unstable housing found moderate to high positive predictive value (range: 0.55–0.95), mixed specificity (range: 0.30–0.92), and low sensitivity (< 0.25) [28, 29, 30].

### 2.3 SCOUTT Exposure

Patient‐level exposure was defined as receipt of health care in a facility that participated in SCOUTT Phase 1. This included the 18 facilities noted above and a small number of affiliated community clinics that received MOUD training from SCOUTT teams. Phase 1 of the SCOUTT intervention started at the end of FY 2018, with some pre‐implementation activities occurring in the middle of 2018. We coded 2016–2018 as pre‐implementation FYs, and 2019–2020 as post‐implementation FYs. To ensure consistent pre‐ and post‐implementation comparisons, we restricted analyses to facilities with at least 1 PEH meeting cohort criteria (described above) during both periods.

### 2.4 Outcomes

The outcome was any MOUD receipt, determined from prescription fills for buprenorphine, buprenorphine/naloxone, long‐acting injectable buprenorphine, and extended‐release injectable naltrexone from VHA pharmacy records; or methadone treatment at a VHA opioid treatment program. We examined “any receipt” of MOUD during each FY, which aligns with VHA performance measure definitions, and receipt of each specific type of MOUD (buprenorphine, naltrexone, methadone) as a secondary outcome. We only included buprenorphine and naltrexone formulations approved for OUD treatment and excluded those offered primarily for pain or alcohol use disorder, respectively.

### 2.5 Covariates

We included covariates that could vary across SCOUTT and non‐SCOUTT facilities and account for MOUD use, encompassing both time‐independent and time‐varying patient‐level characteristics, as well as facility‐ and health‐system‐level factors measured in FY 2018.

#### 2.5.1 Patient‐Level Covariates

Patient‐level covariates included age, race and ethnicity, sex, marital status, geographic residence, and VA priority score. We included race and ethnicity as covariates given well‐documented racial and ethnic differences in OUD treatment access [10], and because the demographic composition of patient populations varies geographically in ways that may differ systematically between SCOUTT and non‐SCOUTT facilities. VA prioritizes self‐report of race and ethnicity, though proxy and staff observation reports also occur [31]. The VA priority score is a composite of income, military service history, and disability that determines VHA health care eligibility and copayment status. To account for clinical comorbidity, we controlled for PEHs' past year NOSOS score, a VHA‐derived comorbidity indicator predictive of healthcare costs [32]. We also controlled for the number of medical conditions from the Elixhauser Comorbidity Index, chronic pain diagnoses, and the presence of behavioral health comorbidities documented by ICD codes: alcohol use disorder, drug use disorder exclusive of OUD, anxiety disorders, depressive disorders, post‐traumatic stress disorder (PTSD), serious mental illnesses, and other mental health disorders (i.e., adjustment, somatic, or personality disorders). To account for the co‐occurrence of several homeless experience indicators, we created a single binary housing status variable (literal homelessness versus unstable housing) using established definitions (Tables S1 and S3) [17].

We also controlled for receipt of services for seven adverse social determinants of health including: psychosocial needs, history of legal involvement, violence exposure, financial difficulties, access barriers, history of military sexual trauma, and lack of social support (Tables S1 and S4). To retain the most relevant information while reducing dimensionality and minimizing potential multicollinearity, we conducted convex logistic principal component analysis and incorporated into the analytic models the first two resultant principal components (PC), PC1 and PC2 reflecting these social determinants of health.

#### 2.5.2 Timing of Patient‐Level Covariates

Some patient covariates, such as medical morbidity, chronic pain, behavioral health factors, mental health conditions, literal homelessness, and social determinants of poor health, were measured concurrently with the exposure. To reduce the potential for bias, we calculated an average of the patient‐level covariates during the pre‐implementation period (FY2016–2018). If pre‐implementation data for a given patient was unavailable, we used values from the first available post‐implementation year in which the patient had a record. This approach strengthened the temporal ordering of covariates and exposure, improving the reliability of our inferences.

#### 2.5.3 Facility and Health System Covariates

For facility context, the controlled characteristics in FY 2018 included: census region, facility type (i.e., medical center versus clinic), urban/rural, presence of an opioid treatment program (i.e., for methadone), and presence of specialized primary care programs for PEH in FY 2018. Health system variables included complexity score, which is based on the presence of teaching and clinical programs and volume of high‐need patients; number of patients diagnosed with OUD (per 1000); and facility performance on MOUD receipt in FY 2018's first quarter.

### 2.6 Statistical Analyses

The primary analyses occurred in four steps. First, we summarized the patient‐, facility‐ and health system‐level characteristics for SCOUTT and non‐SCOUTT groups. We examined patient‐level characteristics separately during the pre‐ and post‐implementation periods. If a PEH met cohort criteria in ≥ 2 study years within the pre‐ or post‐period, we summarized data from the first year in the respective period. Facility‐ and health system‐characteristics were then summarized using facility‐level data. We used cross‐tabulations to examine the distribution of categorical variables, and summarized medians and interquartile ranges (IQRs) for continuous variables. We compared the distribution between SCOUTT and non‐SCOUTT groups, applying the Chi‐squared test or Fisher's exact test for categorical variables and Wilcoxon rank‐sum test for numeric variables as appropriate.

Second, we used line charts to visualize the annual trends in MOUD for the pre‐ and post‐implementation years in SCOUTT and non‐SCOUTT facilities, as one step toward evaluating the suitability of a conventional difference‐in‐differences (DiD) design for patient‐level data. The key requirement for causal inference under DiD is that, in the absence of the intervention, outcome trends in the intervention sites would have been parallel to those in the comparison sites throughout the full study period. Information on trends in pre‐implementation years informed our choice of statistical methods to account for historical trends in facilities volunteering for SCOUTT compared to facilities that did not (below).

Third, we used mixed‐effects quasi‐Poisson regression models with entropy balancing to estimate the Average Treatment Effect on the Treated (ATT) in post‐implementation years. The ATT estimate represents the effect of SCOUTT for PEH receiving care in facilities that participated in SCOUTT, comparing their observed outcomes with those of individuals with similar observed characteristics in non‐SCOUTT facilities. Under the assumption that this comparison represents a valid counterfactual, the ATT reflects the outcomes SCOUTT facility patients would have experienced had their facilities not participated in SCOUTT. Entropy balancing was applied for covariate adjustment; it assigns weights to observations so that key characteristics were exactly balanced (i.e., equal means and proportions) between the SCOUTT and non‐SCOUTT groups [33].

In addition to the patient‐ and facility‐level covariates noted above, we included facility pre‐implementation levels of MOUD. Specifically, we calculated the percent of PEH who received MOUD during the pre‐implementation period in each facility. Averages were calculated separately for each facility in each year, and then the facility‐specific average of the averages was chosen to avoid multicollinearity. This was done separately for each type of MOUD. For all covariates, we calculated the standardized mean differences (SMDs) for each characteristic before and after weighting to document achieved covariate balance.

The quasi‐Poisson approach was used to provide robust standard errors for binary outcomes. The model, run for each type of MOUD, included facility‐ and patient‐level random effects to account for clustering within facilities and repeated observations among patients. However, for injectable naltrexone, we included only the facility random effect due to low usage. For all models, we report marginal effects as absolute differences in the predicted probability of receiving MOUD during the post‐implementation period. These marginal effects were obtained via model‐based standardization, averaging predicted contrasts over the observed covariate distribution to yield adjusted percentage‐point differences.

Finally, as an alternate approach to control for historical trends in MOUD receipt across SCOUTT and non‐SCOUTT facilities, we conducted a synthetic DiD analysis using the facility‐level annual treatment rates during the pre‐ and post‐implementation periods. Synthetic DiD is a quasi‐experimental method to estimate the effect of a policy or program by comparing outcome changes from pre‐ to post‐implementation in the treated group (SCOUTT facilities) with a reweighted control group (non‐SCOUTT facilities) that matches the treated group's pre‐intervention trends [34]. It addresses violations of the parallel trends assumption in conventional DiD by reweighting observations from the comparison group to align with the treated group's pre‐implementation trajectory, thereby generating a counterfactual for post‐implementation comparison. Using this approach, we derived an ATT estimate of the effect of SCOUTT on the facility‐level rate of MOUD among PEH. We calculated the 95% CI for the DiD effect using bootstrapping with 500 replications.

All statistical analyses were conducted using R (version 4.4.1). We used the R packages WeightIt and cobalt for entropy balancing and covariate balance assessment, lme4 for mixed‐effects modeling, emmeans for estimation of marginal effects, and synthdid for synthetic DiD analysis. We considered statistical significance at an alpha level of 0.05.

### 2.7 Sensitivity Analyses

Mixed‐effects models account for clustering within patients and facilities but, in the conventional random‐intercept specification, assume that the facility‐specific random effect is independent of the covariates included in the model, including SCOUTT participation. Entropy balancing reduces confounding attributable to measurable characteristics, but it does not establish or test independence between the latent facility random effect and SCOUTT participation or the other included regressors. Thus, we cannot verify the random‐effects independence assumption directly. As a sensitivity analysis, we re‐estimated the primary outcome models using a facility fixed effects approach without this assumption. Because SCOUTT status is time‐invariant within our post‐implementation‐only analytic window, substituting facility fixed effects for random effects in a single‐stage model would result in exact collinearity between the SCOUTT indicator and the facility fixed effects. We therefore used a two‐stage approach instead. In the first stage, a quasi‐Poisson regression with facility fixed effects was fit, with the same entropy balancing weight applied. We used cluster‐robust standard errors at the patient level to account for within‐patient correlation across fiscal years. In the second stage, facility‐specific predicted rates for SCOUTT and non‐SCOUTT groups were aggregated using the inverse standard error of the log‐scale facility‐specific estimates to obtain the ATT estimate.

A potential concern with synthetic DiD is regression to the mean (RTM), whereby temporarily elevated pre‐period rates in upweighted non‐SCOUTT facilities subsequently revert, inflating the apparent treatment effect [35, 36]. To assess the potential impact of this bias, we conducted a sensitivity analysis excluding facilities most susceptible to regression to the mean symmetrically from both SCOUTT and non‐SCOUTT groups. Specifically, we excluded facilities with extreme pre‐implementation slopes and those whose slope may not be reliable due to sparse data. Exclusion criteria were defined for this sensitivity analysis using the IQR rule applied within each group: facilities whose pre‐period quarterly MOUD treatment rate slope exceeded 1.5 × IQR above the upper quartile of their group's slope distribution (FY2016–2018), and facilities in the bottom 10th percentile of total patient‐fiscal year observations during the pre‐implementation period.

## 3 Results

### 3.1 Patient Characteristics and Facility Context in SCOUTT and Non‐SCOUTT Facilities

There were 30 SCOUTT and 811 non‐SCOUTT facilities classified as of the Phase 1 implementation period. A total of 20,746 unique PEH received care during the post‐implementation years (FY2019–2020), with 2464 in SCOUTT facilities and 18,282 in non‐SCOUTT facilities (Table 1). PEH in SCOUTT facilities were less likely to reside in rural areas (12.9% vs. 19.9%), or to have a diagnosis of alcohol use disorder (51.2% vs. 57.0%), or other mental health disorders (22.0% vs. 26.8%). All other standardized mean differences in the post‐implementation period were less than or equal to 0.10. Comparing the overall pre‐implementation period and post‐implementation period (Table 1 and Table S2), the characteristics of PEH were similar over time.

**TABLE 1:** Patient characteristics in the post‐implementation period in SCOUTT and non‐SCOUTT facilities (fiscal years 2019–2020).

Characteristic	Overall (*N* = 20,746)	SCOUTT (*N* = 2464)	Non‐SCOUTT (*N* = 18,282)	SMD^a^
Age in years				—
18–34	3497 (16.9%)	396 (16.1%)	3101 (17.0%)	−0.02
35–49	5047 (24.3%)	554 (22.5%)	4493 (24.6%)	−0.05
50–64	9059 (43.7%)	1080 (43.8%)	7979 (43.6%)	0.004
65+	3143 (15.1%)	434 (17.6%)	2709 (14.8%)	0.08
Male sex	19,118 (92.2%)	2250 (91.3%)	16,868 (92.3%)	−0.04
Race and ethnicity				—
Non‐Hispanic White	12,578 (60.6%)	1505 (61.1%)	11,073 (60.6%)	0.01
Non‐Hispanic Black	5756 (27.7%)	694 (28.2%)	5062 (27.7%)	0.01
Hispanic	1216 (5.9%)	141 (5.7%)	1075 (5.9%)	−0.007
Other	1196 (5.8%)	124 (5.0%)	1072 (5.9%)	−0.04
Marital status				—
Married	3769 (18.2%)	438 (17.8%)	3331 (18.2%)	−0.01
Single	5946 (28.7%)	691 (28.0%)	5255 (28.7%)	−0.02
Previously married	10,343 (49.9%)	1244 (50.5%)	9099 (49.8%)	0.01
Missing	688 (3.3%)	91 (3.7%)	597 (3.3%)	0.02
Rural	3959 (19.1%)	317 (12.9%)	3642 (19.9%)	−0.19
VA priority group				
Service‐connected disability	11,828 (57.0%)	1425 (57.8%)	10,403 (56.9%)	0.02
Very low income	7779 (37.5%)	893 (36.2%)	6886 (37.7%)	−0.03
No service connection, copay required	918 (4.4%)	121 (4.9%)	797 (4.4%)	0.03
Other	221 (1.1%)	25 (1.0%)	196 (1.1%)	−0.006
Literal homelessness^b^	16,004 (77.1%)	1847 (75.0%)	14,157 (77.4%)	−0.06
Adverse SDOH^c^, median (IQR)	5.0 (1.0–5.0)	5.0 (1.0–5.0)	5.0 (1.0–5.0)	0.02
Prior year NOSOS score^d^				—
High	3452 (16.6%)	419 (17.0%)	3033 (16.6%)	0.01
Medium	12,163 (58.6%)	1486 (60.3%)	10,677 (58.4%)	0.04
Low	4524 (21.8%)	491 (19.9%)	4033 (22.1%)	−0.05
Missing	607 (2.9%)	68 (2.8%)	539 (2.9%)	−0.01
Medical conditions, median (IQR)	2.0 (1.0–4.0)	2.0 (1.0–4.0)	2.0 (1.0–3.0)	0.03
Pain condition diagnosis	12,703 (61.2%)	1530 (62.1%)	11,173 (61.1%)	0.02
Mental health and substance use disorder diagnoses				
Drug use disorder	14,891 (71.8%)	1726 (70.0%)	13,165 (72.0%)	−0.04
Alcohol use disorder	11,688 (56.3%)	1261 (51.2%)	10,427 (57.0%)	−0.12
Depression	12,694 (61.2%)	1536 (62.3%)	11,158 (61.0%)	0.03
Anxiety	8200 (39.5%)	926 (37.6%)	7274 (39.8%)	−0.05
PTSD	10,016 (48.3%)	1296 (52.6%)	8720 (47.7%)	0.10
Serious mental illness	4852 (23.4%)	583 (23.7%)	4269 (23.4%)	0.007
Other	5433 (26.2%)	541 (22.0%)	4892 (26.8%)	−0.11

Abbreviations: IQR, interquartile range; PTSD, post‐traumatic stress disorder; SCOUTT, stepped care for opioid use disorder train the trainer initiative; SDOH, social determinants of health; SMD, standardized mean difference; VA, Veterans Affairs.

^a^ For post‐implementation comparisons, SMDs were used as a metric of covariate balance.

^b^ Met study criteria for literal homelessness, compared to unstable housing. Evidence of literal homelessness includes an International Classification of Disease (ICD) version 10 diagnosis of homelessness; negative response to the question “In the past two months, have you been living in stable housing that you own, rent, or stay in as part of a household?”; residence in VA transitional housing; or receipt of community outreach, employment assistance or healthcare designated for homeless Veterans. Evidence of unstable housing includes ICD‐10 diagnosis of inadequate housing or unstable housing among the housed; living in subsidized housing for formerly homeless veterans; or an affirmative response to the question “Are you worried or concerned that in the next two months you may not have stable housing that you own, rent, or stay in as part of a household?” Administrative data definitions provided in Table S1.

^c^ Documented by ICD codes, structured text in clinical notes, or receipt of VA programs or services relating to (a) legal involvement; (b) exposure to violence; (c) military sexual trauma; (d) financial difficulties; (e) access to care concerns; (f) lack of social support; and (g) other unspecified psychosocial needs. Administrative data definitions provided in Table S1.

^d^ NOSOS is a VA‐derived risk adjustment score that predicts VA health care costs based on diagnosis‐based hierarchical condition categories, pharmacy data, and demographic characteristics. Scores are centered so that 1.0 reflects the costs of a typical VA patient. We categorized log‐transformed NOSOS scores as low (< 0.0), medium (0.0–2.0) and high (≥ 2.0).

In FY 2018, SCOUTT facilities were disproportionately concentrated in urban areas (93.3% of SCOUTT and 66.8% of non‐SCOUTT facilities were in urban settings, Table 2). Facilities that participated in SCOUTT were more likely, compared to other facilities, to be medical centers (53.3% vs. 16.3%), operate in a high complexity health system (63.3% vs. 36.0%), have specialized primary care programs for PEH (33.3% vs. 5.8%), and operate opioid treatment programs (20.0% vs. 3.1%). In addition, SCOUTT facilities outperformed other facilities on the VHA MOUD performance measure (percentage of diagnosed patients receiving MOUD: 40.5% vs. 31.6%) in FY 2018.

**TABLE 2:** Facility characteristics in SCOUTT and non‐SCOUTT facilities, fiscal year 2018.

Characteristic	Facilities, *n* (%)			SMD^a^
	Overall (*N* = 841)	SCOUTT (*N* = 30)	Non‐SCOUTT (*N* = 811)	
Census region				—
West	172 (20.5%)	10 (33.3%)	162 (20.0%)	0.31
Midwest	199 (23.7%)	5 (16.7%)	194 (23.9%)	−0.18
South	321 (38.2%)	12 (40.0%)	309 (38.1%)	0.04
Northeast	149 (17.7%)	3 (10.0%)	146 (18.0%)	−0.23
Medical center	148 (17.6%)	16 (53.3%)	132 (16.3%)	0.84
Urban	570 (67.8%)	28 (93.3%)	542 (66.8%)	0.70
Has methadone treatment program^b^	31 (3.7%)	6 (20.0%)	25 (3.1%)	0.55
Health system complexity^c^				—
High	311 (37.0%)	19 (63.3%)	292 (36.0%)	0.57
Medium‐high	261 (31.0%)	8 (26.7%)	253 (31.2%)	−0.10
Medium	129 (15.3%)	0 (0.0%)	129 (15.9%)	−0.62
Low	140 (16.6%)	3 (10.0%)	137 (16.9%)	−0.20
Has an HPACT^d^	57 (6.8%)	10 (33.3%)	47 (5.8%)	0.74
OUD diagnoses per 1000 patients^e^, Median (IQR)	11.0 (8.4–14.4)	11.3 (10.5–12.0)	10.9 (8.2–14.4)	0.03
Percentage of patients diagnosed with OUD who received any MOUD^e^, Median (IQR)	31.9 (24.1–42.6)	40.5 (33.5–44.4)	31.6 (24.1–42.6)	0.54

Abbreviations: HPACT, homeless patient aligned care teams; IQR, interquartile range; MOUD, medication treatment for opioid use disorder; OUD, opioid use disorder; SCOUTT, stepped care for opioid use disorder train the trainer initiative; SMD, standardized mean difference.

^a^ SMDs were used as a metric of covariate balance.

^b^ Refers to a federally qualified opioid treatment program certified to provide methadone.

^c^ Classification of VA facilities based on patient characteristics and the breadth of clinical and educational programs offered at the facility. High (1a)—facilities with highest volume, high risk patients, large number of complex clinical programs, and large research and teaching programs; Medium‐high (1b, 1c)—facilities with medium‐high volume, medium and high risk patients, some complex clinical programs, and medium to large research and teaching programs; Medium (2)—facilities with medium patient volume, low risk patients, few complex clinical programs, and no research or teaching programs; Low (3)—facilities with low volume, low risk patients, few or no complex clinical programs, and no research or teaching programs.

^d^ Refers to specialized primary care model for Veterans with experience of homelessness.

^e^ Estimates derived from data among all patients who received health care in the health system (i.e., medical center and affiliated outpatient clinics) in Quarter 1 of Fiscal Year 2018.

For each of the patient and facility characteristics, absolute unweighted SMDs ranged from 0.0 to 0.84 with the largest difference observed in facility type (i.e., medical center versus community‐based outpatient clinic; Tables 1 and 2). We confirmed, after weighting, that all SMDs equaled zero as required in entropy balancing (Figure S1A–D).

### 3.2 Pre‐ and Post‐Implementation Patterns of MOUD Among PEH

From 2016 to 2020, the percentage of PEH receiving MOUD increased across both SCOUTT and non‐SCOUTT facilities, with the greatest increase for buprenorphine (Figure 1). In the pre‐implementation period, any MOUD receipt was higher and rose faster in SCOUTT facilities compared to non‐SCOUTT facilities. After implementation, both types of facilities continued to increase MOUD provision. Buprenorphine increased over the study period, while methadone and naltrexone tended to decline or remain stable.

**FIGURE 1:**
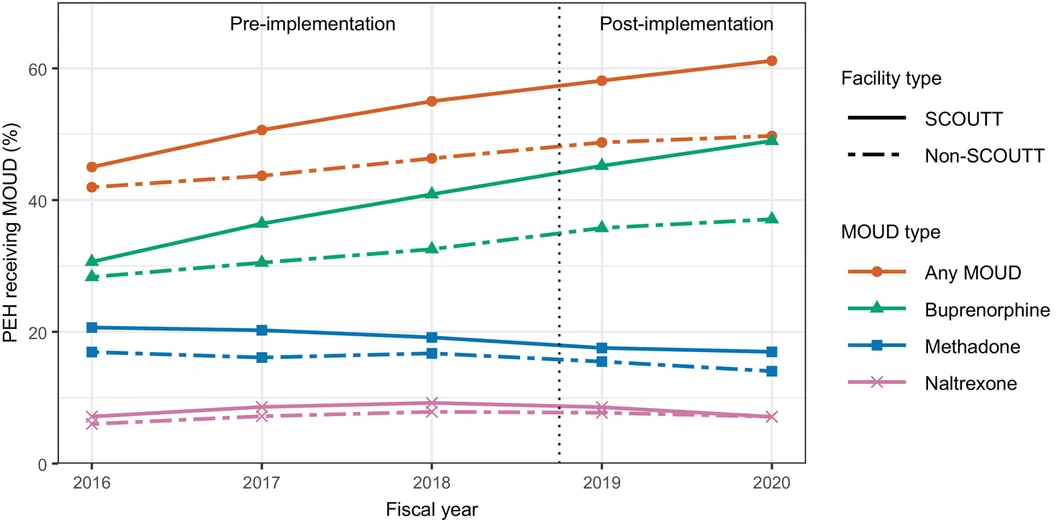
Temporal trends in the percentage of patients with homeless experience receiving medication treatment for opioid use disorder, by type of facility and type of medication. MOUD, medication treatment for opioid use disorder; PEH, Persons experiencing homelessness; SCOUTT, stepped care for opioid use disorder train the trainer initiative. Lines show the unadjusted annual percentage of PEH with an OUD diagnosis and ≥ 1 primary care visit who received each type of MOUD in each fiscal year, FY2016–2020. Solid lines (in each color) indicate achieved medication treatment rates for facilities participating in the SCOUTT initiative. Dot‐dash lines indicate non‐SCOUTT facilities. The vertical dotted line indicates the time of SCOUTT implementation.

### 3.3 Estimated ATT for the Probability of Receiving MOUD in the Post‐Implementation Period (FY2019‐2020)

During the post‐implementation period, receiving care in a SCOUTT facility was associated with significantly higher probability of receiving any MOUD among PEH. Specifically, the estimated ATT indicated a 6‐percentage point increase in the probability of receiving any MOUD (Figure 2; risk difference (RD) = 6.16, 95% CI: 2.33–9.99). For buprenorphine MOUD, the risk difference was 7.16 percentage points (95% CI: 4.35–9.97). There were no statistically significant differences in MOUD for methadone (RD = −0.19, 95% CI: −1.03–0.65) or naltrexone (RD = 0.10, 95% CI: −1.85–2.05). In the facility fixed effects sensitivity analysis, findings were consistent with the primary mixed effects results (Figure S2).

**FIGURE 2:**
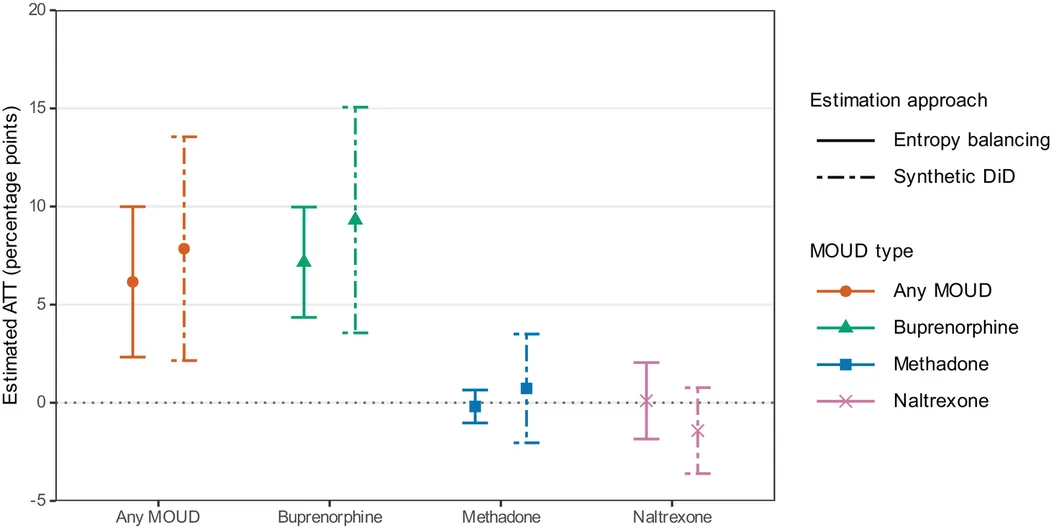
Estimated effect of the stepped care for opioid use disorder train the trainer intervention on receipt of medication treatment for opioid use disorder, among patients with homeless experience. ATT, Average treatment effect on the treated; DiD, difference‐in‐differences; FY, fiscal year; MOUD, medication treatment for opioid use disorder; PEH, Persons experiencing homelessness; SCOUTT, stepped care for opioid use disorder train the trainer initiative. Points represent ATT estimates, and vertical bars indicate 95% confidence intervals. Estimates are expressed as percentage‐point differences in MOUD receipt, calculated for primary care PEH who received care in SCOUTT facilities compared with the counterfactual scenario in which the same facilities had not participated in SCOUTT. The horizontal dotted line at 0 denotes the null ATT. Solid lines (in each color) and dot‐dash lines represent two distinct methods for calculating ATTs to account for historical trends in MOUD prescribing. (1) The solid line reflects the entropy balancing approach. This calculates differences in MOUD for PEH in SCOUTT facilities, compared to the counterfactual scenario, calculated in the post‐implementation years only (FY2019–2020). ATTs were estimated using mixed‐effects quasi‐Poisson regression models with entropy balancing to adjust for patient‐ and facility‐level covariates, including facility‐level rates of MOUD in the pre‐implementation years (FY2016–2018). (2) The dot‐dash line reflects calculation of ATT from a synthetic DiD estimate. This represents the difference in the percentage of PEH in a facility who received MOUD, calculated as the percent among PEH in SCOUTT facilities minus the expected percent who would receive MOUD under the counterfactual scenario, in the post‐implementation years (FY2019–2020) minus the pre‐implementation years (FY2016–2018). 95% confidence intervals were estimated via bootstrapping with 500 replications. In the entropy balancing approach, *p*‐values for any MOUD, buprenorphine, methadone, and naltrexone estimates were 0.002, < 0.001, 0.66, and 0.92, respectively. In synthetic DiD, no *p*‐values were obtained.

### 3.4 Estimated ATT From Synthetic DiD Analysis for the Percentage of PEH Receiving MOUD in SCOUTT Facilities Compared to Matched Synthetic Comparison Facilities, From Pre‐ to Post‐Implementation Periods (FY2019‐2020 vs. FY2016‐2018)

The synthetic DiD analysis further estimated the causal effect of SCOUTT implementation by comparing post‐ vs. pre‐implementation trends between SCOUTT and a matched synthetic control group. The ATT indicated that PEH in SCOUTT facilities experienced a 7.85 percentage point increase in the annual rate of MOUD receipt (Figure 2; 95% CI: 2.15–13.55), compared to how their outcomes would have changed in the absence of the program. For buprenorphine MOUD specifically, the estimated ATT was 9.31 (95% CI: 3.56–15.06), suggesting that SCOUTT implementation led to a substantial improvement in access to this treatment. Findings for methadone (DiD, 0.73; 95% CI: −2.04–3.50) and naltrexone (DiD, −1.42; 95% CI: −3.61–0.77) were consistent with the primary analysis, showing no statistically significant SCOUTT‐associated effects. Observed and counterfactual MOUD treatment trends underlying the synthetic DiD estimates are shown in Figure S3.

To assess potential regression to the mean bias, the sensitivity analysis excluded 4 of 30 SCOUTT facilities (13.3%) and 95 of 811 non‐SCOUTT facilities (11.7%) with extreme pre‐period slopes or small sizes. Findings were consistent with the primary synthetic DiD estimates, with SCOUTT implementation remaining associated with statistically significant increases in any MOUD and buprenorphine MOUD receipt (Figures S4 and S5). Given that methadone and naltrexone showed no significant effect in the main analysis, this sensitivity analysis focused solely on the any MOUD and buprenorphine outcomes.

## 4 Discussion

It is important that programs designed to expand MOUD provision succeed in reaching populations at elevated risk of suffering OUD harms [37]. Within a national VHA initiative to expand MOUD provision in low‐intensity office‐based settings, we examined MOUD delivery among primary care patients with experience of homelessness. We found overall increases in the percentage of diagnosed PEH receiving MOUD, especially in VHA facilities that received stepped‐care training and implementation support. The probability of MOUD receipt among PEH seen in SCOUTT facilities was 6 percentage points higher post‐implementation than for PEH seen in non‐SCOUTT facilities. MOUD increases were driven by buprenorphine, the focus of SCOUTT facilitation efforts. Our findings suggest implementation strategies to expand MOUD in low‐intensity office‐based settings, such as primary care and general mental health clinics, could be effective for PEH for whom specialized addiction treatment settings might be less desirable.

This study offers insight on the impact of stepped‐care approaches to OUD treatment for patients from vulnerable populations. The increases in buprenorphine are consistent with two prior evaluations of the VHA's SCOUTT initiative that showed increases in the number of providers prescribing buprenorphine MOUD over time [20, 38], and greater increases in the number of buprenorphine‐treated patients per provider in facilities that received SCOUTT facilitation compared to VHA facilities that lacked SCOUTT participation [20]. However, the prior evaluations did not focus on impacts among PEH specifically. That we observed similar improvements for homeless‐experienced patients is notable. The stepped‐care initiative was not designed to address the unique barriers among PEH such as housing instability, discrimination, and negative experiences with care in traditional environments. And yet, SCOUTT's education, training, and learning collaborative may have increased provider comfort in prescribing buprenorphine for patients with adverse social determinants of health. We found MOUD gains for PEH were achieved despite SCOUTT facilities serving older, more clinically complex populations with higher rates of chronic pain and PTSD. The deployment in urban, high‐resource facilities, some of which had pre‐existing addiction care infrastructure, may have contributed as well.

One strength of this study was the application of multiple statistical methods to obtain valid inferences concerning the impact of the stepped‐care model on MOUD. We found that facilities that took part in SCOUTT had higher overall performance on measures of MOUD access and greater increases in MOUD among PEH in FY2016–2018, suggesting that changes in provider acceptance or motivation to treat OUD were already underway in those facilities. Providers who participated in SCOUTT likely had strong baseline knowledge of MOUD [25] and a desire to provide high quality of care for persons with OUD. To ensure comparability of SCOUTT and non‐SCOUTT facilities, we included facility‐level covariates, applied two quasi‐experimental methods of weighting observations at the patient‐ and facility‐levels to improve covariate balance and assess robustness of model assumptions. Our finding that increases in MOUD within SCOUTT facilities were driven by buprenorphine, with no increase in other MOUD types, reinforces clinical face validity, as implementation of buprenorphine is the focus of SCOUTT program support. Taken together, the medication‐specific results and consistency of study findings when applying two quasi‐experimental designs lend confidence to our study's conclusions.

We note limitations. First, we were unable to measure the extent of exposure to SCOUTT trainings and implementation support within and across facilities. Clinical teams varied in their participation in SCOUTT trainings and implementation support, and there could have been cross‐contamination of SCOUTT information from participating to non‐participating facilities. Second, there could be unique characteristics of the clinical teams who volunteered for SCOUTT; those may not be shared among providers under usual care in non‐SCOUTT facilities. By design, the ATT estimate reflects increases in MOUD that would be expected among facilities that chose to participate in SCOUTT, relative to how those same facilities would have performed in the absence of participation, under the assumption that the weighted comparison facilities provide a valid counterfactual. Although entropy balancing achieved exact balance on measured patient‐ and facility‐level characteristics and pre‐implementation MOUD rates, the assumption cannot be verified, and residual confounding by unmeasured facility characteristics remains possible.

Third, the mixed‐effects estimates rely on the assumption that the facility‐specific random effect is independent of the included covariates, including SCOUTT participation. This is a strong assumption that is not directly testable. However, comparable estimates between the random effect model and the fixed‐effect sensitivity analysis suggest plausibility. The similar synthetic DiD estimates provide reassurance under a different design and set of assumptions but do not eliminate this limitation.

Fourth, the cohort focused on Veterans with homeless experience obtaining VHA primary care. Because SCOUTT was implemented in primary care, pain and mental health settings, our findings could miss programmatic effects for PEH not accessing primary care. However, we expect this possibility to be small as patients diagnosed with OUD often have high rates of contact with primary care [39]. Additionally, findings are limited to PEH with evidence of homelessness or unstable housing in their medical record. Given that homelessness and other adverse social determinants of health are under‐documented [40], findings may not generalize to PEH who are not receiving VHA‐provided housing assistance and whose unhoused experience would not be known to the VHA. Our measure of MOUD includes a single pharmacy fill or visit to an opioid treatment program and may not always reflect long‐term engagement [27]. Finally, our measure of MOUD only included medications filled or dispensed within the VHA. Given that a small percentage of treated Veterans receive MOUD from contracted providers outside the VHA (< 2% in FY 2015–2020) [41], estimates of MOUD receipt among PEH in the present study are likely conservative.

## 5 Conclusions

National efforts to advance a stepped‐care model of OUD with emphasis on MOUD initiation in low‐intensity office‐based settings are succeeding in expanding capacity to treat patients with OUD, including PEH. Further support of SCOUTT programming in the VHA's homeless‐tailored primary care settings could further improve treatment opportunities for PEH with OUD [27]. Lessons from the VHA could inform practices to implement stepped‐care approaches in State Medicaid programs, community mental health clinics, Federally Qualified Health Centers, and other office‐based settings that serve PEH and other underserved populations.

## Funding

This material is based upon work supported by the US Department of Veterans Affairs Health Services Research & Development (CDA 19‐233, Award No IK2HX003090) and Quality Enhancement Research Initiative (EBP 22‐109). This investigation was supported by the University of Utah Translational Research: Implementation, Analysis, & Design team, with funding in part from the National Center for Research Resources, National Institutes of Health and the National Center for Advancing Translational Sciences, National Institutes of Health, through Grant UM1TR004409 (formerly 5UL1TR001067‐05, 8UL1TR000105 and UL1RR025764). This work further received institutional support from the Program for Addiction Research, Clinical Care, Knowledge, and Advocacy (PARCKA) and Greater Intermountain Node (GIN), funded in part from the National Institute of Drug Abuse, National Institutes of Health (UG1DA049444‐01) at the University of Utah, and VA Salt Lake City Health Care System's Informatics, Decision‐Enhancement, and Analytic Sciences (IDEAS) Center of Innovation (CIN 13‐414).

## Disclosure

Views expressed in this manuscript are those of the authors and do not represent the positions or views of the United States Department of Veterans Affairs, National Institutes of Health, or the United States Government.

## Conflicts of Interest

All authors were employed or contracted by the Department of Veterans Affairs during the preparation of this manuscript. Author Adam J. Gordon receives a royalty from Wolters‐Kluwer for an online chapter on alcohol management in the perioperative period from the UpToDate online reference (unrelated to this manuscript). In the last 3 years, author Adam J. Gordon has been on the board of directors for the American Society of Addiction Medicine (ASAM), the Association for Multidisciplinary Education and Research in Substance use and Addiction (AMERSA), and the International Society of Addiction Journal Editors (ISAJE), all nonprofit organizations (he receives no remuneration for these duties). He receives remuneration for his duties as Editor‐in‐Chief for AMERSA's peer reviewed journal. Author Stefan G. Kertesz holds stock in two health care companies, DexCom and Thermo Fisher, and receives a royalty from Wolters‐Kluwer for an online chapter on homeless health care in the UpToDate online reference. In the last 3 years, author Sophia Huebler had a financial relationship with ARCA Biopharma, in which she received payments for consultation work with a prior employer. The remaining authors declare no conflicts of interest.

## Supporting information


**Table S1:** Study Definitions from Veterans Health Administration (VHA) Administrative and Electronic Health Records.
**Table S2:** Patient Characteristics in the Pre‐Implementation Period in SCOUTT and non‐SCOUTT Facilities (Fiscal Years 2016–2018).
**Table S3:** Homeless Experience Indicators, by Time (Pre‐Implementation versus Post‐Implementation) and Type of Facility (SCOUTT versus Non‐SCOUTT).
**Table S4:** Social Determinant of Health Indicators, by Time (Pre‐Implementation versus Post‐Implementation) and Type of Facility (SCOUTT versus non‐SCOUTT).
**Figure S1A:** Covariate Balance between SCOUTT and Non‐SCOUTT Groups before and after Weighting for the Outcome of Receiving MOUD Treatment.
**Figure S1B:** Covariate Balance between SCOUTT and Non‐SCOUTT Groups before and after Weighting for the Outcome of Receiving Buprenorphine Treatment.
**Figure S1C:** Covariate Balance between SCOUTT and Non‐SCOUTT Groups before and after Weighting for the Outcome of Receiving Methadone Treatment.
**Figure S1D:** Covariate Balance between SCOUTT and Non‐SCOUTT Groups before and after Weighting for the Outcome of Receiving Naltrexone Treatment.
**Figure S2:** Estimated SCOUTT Effect on the Probability of Receiving Medication Treatment for Opioid Use Disorder: Primary Mixed Effects and Facility Fixed Effect Sensitivity Analysis.
**Figure S3:** Trends in Outcomes before and after SCOUTT Implementation Using Synthetic DiD.
**Figure S4:** Estimated SCOUTT Effect on the Probability of Receiving Medication Treatment for Opioid Use Disorder: Primary and Sensitivity Analysis of Synthetic DiD.
**Figure S5:** Synthetic DiD Trends After Excluding Facilities with Extreme Pre‐Implementation Slopes and Small Samples.

## Data Availability

Access to a limited, deidentified dataset can be made available pending ethical approval and in accordance with VA guidelines. Those wishing to access the study data may contact the corresponding author to discuss the VA data access approval process.
