# Supplementary material for: Impact of a National Stepped‐Care Model of Opioid Use Disorder Treatment on Access to Medication Therapies Among Homeless‐Experienced Veterans
Source: Health Serv Res. 2026 Oct 13;61(5):e70171. doi: 10.1111/1475-6773.70171 (PMC13661296; doi:10.1111/1475-6773.70171)
Supplement: Supplementary file 1 [file HESR-61-e70171-s001.docx]

**Title:** Impact of a National Stepped-Care Model of Opioid Use Disorder Treatment on Access to Medication Therapies among Homeless-Experienced Veterans

**supplementary Material**

Table S1. Study Definitions from Veterans Health Administration (VHA) Administrative and Electronic Health Records

Table S2. Patient Characteristics in the Pre-Implementation Period in SCOUTT and non-SCOUTT Facilities (Fiscal Years 2016-2018)

Table S3. Homeless Experience Indicators, by Time (Pre-Implementation versus Post-Implementation) and Type of Facility (SCOUTT versus Non-SCOUTT)

Table S4. Social Determinant of Health Indicators, by Time (Pre-Implementation versus Post-Implementation) and Type of Facility (SCOUTT versus non-SCOUTT)

Figure S1-A. Covariate Balance between SCOUTT and Non-SCOUTT Groups before and after Weighting for the Outcome of Receiving MOUD Treatment

Figure S1-B. Covariate Balance between SCOUTT and Non-SCOUTT Groups before and after Weighting for the Outcome of Receiving Buprenorphine Treatment

Figure S1-C. Covariate Balance between SCOUTT and Non-SCOUTT Groups before and after Weighting for the Outcome of Receiving Methadone Treatment

Figure S1-D. Covariate Balance between SCOUTT and Non-SCOUTT Groups before and after Weighting for the Outcome of Receiving Naltrexone Treatment

Figure S2. Estimated SCOUTT Effect on the Probability of Receiving Medication Treatment for Opioid Use Disorder: Primary Mixed Effects and Facility Fixed Effect Sensitivity Analysis

Figure S3. Trends in Outcomes before and after SCOUTT Implementation Using Synthetic DiD

Figure S4. Estimated SCOUTT Effect on the Probability of Receiving Medication Treatment for Opioid Use Disorder: Primary and Sensitivity Analysis of Synthetic DiD

Figure S5. Synthetic DiD Trends After Excluding Facilities with Extreme Pre-Implementation Slopes and Small Samples

**Table S1. Study Definitions from Veterans Health Administration (VHA) Administrative and Electronic Health Records**

| **Variable** | **Type of VHA Data** | **Criteria** |
| --- | --- | --- |
| Primary care visit | Clinic location code | Primary location = (170, 171, 172, 173, 174, 175, 176, 177, 178, 156, 157, 322, 323, 338, 350, 348, 534, or 539) AND secondary location is not (107, 115, 152, 311, 333, 334, 999, 474, 430, 328, 321, 329, 435, 103, 147, 148, 169, 181, 182, 199, 216, 221, 224, 229, 324, 325, 424, 425, 428, 444, 445, 446, 447, 448, 449, 450, 527, 528, 530, 536, 537, 542, 545, 546, 579, 584, 597, 611, 686, or nothing);  Or  Secondary location = (179, 322, 323, 350, 531, or 534) AND primary location is not (107, 115, 152, 311, 333, 334, 999, 474, 430, 328, 321, 329, 435, 103, 147, 148, 169, 181, 182, 199, 216, 221, 224, 229, 324, 325, 424, 425, 428, 444, 445, 446, 447, 448, 449, 450, 527, 528, 530, 536, 537, 542, 545, 546, 579, 584, 597, 611, or 686);  or  Primary location=224 AND secondary location =323;  Primary location =326 AND secondary location =350; or  Primary location =527 AND secondary location =534 |
| Opioid use disorder | 1+ diagnosis in inpatient or outpatient setting | International Classification of Disease version 10 (ICD10) = F11.121, F11.122, F11.120, F11.129, F11.14, F11.150, F11.151, F11.159, F11.181, F11.182, F11.188, F11.19, F11.10, F11.222, F11.221, F11.220, F11.229, F11.24, F11.250, F11.251, F11.259, F11.281, F11.282, F11.288, F11.29, F11.23, F11.20 |
| **Literal homelessness*** | | |
| Homeless diagnosis | 1+ diagnosis in inpatient or outpatient setting | ICD10 = Z59.0 |
| Homeless screen | Clinical reminder screening response | “No” response to question - *In the past two months, have you been living in stable housing that you own, rent, or stay in as part of a household?* |
| Health Care for Homeless Veteran program | Clinic location code | 508, 528, 529 |
| Community outreach | Clinic location code | 590 |
| Grant & per diem | Clinic location code | 504, 511 |
| Transitional residence programs | Inpatient treating specialty | 28-29, 37, 39 |
| Employment assistance for homeless Veterans | Clinic location code | 555, 556 |

**Table S1. Study Definitions from Veterans Health Administration (VHA) Administrative and Electronic Health Records**

| **Variable** | **Type of VHA Data** | **Criteria** |
| --- | --- | --- |
| **Unstable housing** | | |
| Unstable housing diagnosis | 1+ diagnosis in inpatient or outpatient setting | ICD10 = Z59.1, Z59.81 |
| Unstable housing screen | Clinical reminder screening response** | “Yes” response to question - *Are you worried or concerned that in the next two months you may NOT have stable housing that you own, rent, or stay in as part of a household?* |
| Permanent supportive housing | Clinic location code | 507, 522, 530 |
| **Behavioral health comorbidities** | | |
| Alcohol use disorder | 1+ diagnosis in inpatient or outpatient setting | ICD10 = F10.1x, F10.2x |
| Drug use disorder other than OUD | 1+ diagnosis in inpatient or outpatient setting | ICD10 = F12.1x, F12.2x, F13.1x, F13.2x, F14.1x, F14.2x, F15.1x, F15.2x, F16.1x, F16.2x, F18.1x, F18.2x, F19.1x, F19.2x |
| Anxiety disorders | 2+ outpatient diagnoses or 1+ inpatient diagnosis | ICD10 = F40.x, F41.x, F42.x, F45.22, F63.3, F93.0, F94.0 |
| Depressive disorders | 2+ outpatient diagnoses or 1+ inpatient diagnosis | ICD10 = F06.3x, F32.x, F33.x, F34.1 |
| Post-traumatic stress disorder | 2+ outpatient diagnoses or 1+ inpatient diagnosis | ICD10 = F43.10, F43.11, F43.12 |
| Serious mental illness | 2+ outpatient diagnoses or 1+ inpatient diagnosis | ICD10 = F31.x, F06.0, F06.2, F20.x, F22, F23, F24, F25.x, F28, F29, F53, F53.1 |
| Other mental health disorders | 2+ outpatient diagnoses or 1+ inpatient diagnosis | ICD10 = F43.0, F43.2x, F43.8, F43.9, F44.x, F48.1, F94.1, F94.2,  F45.0, F45.1, F45.20, F45.21, F45.29, F45.41, F45.42, F45.8, F45.9, F68.A, F68.10, F68.11, F68.12, F68.13, F07.0, F21, F60.x, F68.8, F69 |
| Pain diagnoses related to the back, neck or other joints, neuropathy, migraine, or fibromyalgia | 2+ outpatient diagnoses or 1+ inpatient diagnosis | ICD10 = D86.87, F45.42, G44.209, G89.3, G89.4, M08.1, M25.5x, M25.78, M43.8x, M43.9, M45.0-M45.9, M46.1, M46.46-M46.49, M46.9x, M47.1x, M47.2x, M47.81x, M47.89x, M47.9, M48.0x, M48.1x, M48.8x, M48.9, M50.0x, M50.1x, M50.2x, M51.25-M51.27, M51.34-M51.37, M51.44-M51.47, M51.9, M53.2X7-M53.2X9, M53.3, M53.8x, M53.9, M54.0x- M54.4x, M54.5, M54.6, M54.89, M54.9, M60.80x-M60.831, M60.85x-M60.89, M60.9, M62.830, M72.9, M79.0, M79.2, M79.60x-M79.63x, M79.65x-M79.67x, M79.7, M96.1, M99.31, M99.51, M99.53, M99.61, M99.71, M99.73, M99.79, S39.012D, S39.92XA |
| **Adverse social determinants of health***** | | |
| History of legal involvement | Clinic location code | 591 or 592 |
|  | 1+ diagnosis in inpatient or outpatient setting | ICD10 = Z65.0, Z65.1, Z65.2, Z65.3, Z65.4, Y92.14 |
|  | Health factor text | Presenting issue - legal |
| Exposure to violence | 1+ diagnosis in inpatient or outpatient setting | ICD10 = O9A.3-5, T74, T76, X92, X93, X94, X95, X96, X97, X98, X99, Y00, Y01, Y02, Y03, Y07, Y08, Y09, Y35, Y36, Y37, Y38, Z04.4, Z04.7, Z04.81, Z65.5, Z69, Z91.4 |

**Table S1. Study Definitions from Veterans Health Administration (VHA) Administrative and Electronic Health Records**

| **Variable** | **Type of VHA Data** | **Criteria** |
| --- | --- | --- |
|  | Health factor text | Presenting issue - abuse |
| Military sexual trauma | Clinic location code | 524 |
|  | Clinical reminder screening response | Military sexual trauma screen - yes; |
| Financial difficulties | Clinic location code | 208, 222, 568, 574, 555, 535 |
|  | 1+ diagnosis in inpatient or outpatient setting | ICD10 = Z56, Z59.4, Z59.5, Z59.6, Z59.7, Z59.8, Z59.9 |
|  | Clinical reminder screening response | Economic concerns;  Income source - unemployment benefits;  Income source - public assistance;  Presenting issue - financial |
| Access barriers | Clinical reminder screening response | Access to care concerns;  Presenting issue - transportation |
| Lack of social support | 1+ diagnosis in inpatient or outpatient setting | ICD10 = Z59.2, Z59.3, Z55, Z60, Z62, Z63 |
|  | Health factor text | Social support concerns |
| Other psychosocial needs | 1+ diagnosis in inpatient or outpatient setting | ICD10 = Z65.8, Z65.9 |

* The homeless indicators were combined into a single variable (any evidence of literal homelessness vs. evidence of unstable housing only) to address multicollinearity.

** Response to short survey administered following a clinical reminder. Responses are stored in the Health Factors domain in the VA Corporate Data Warehouse.

*** Codes adapted from: Blosnich JR, Montgomery AE, Dichter ME, et al. Social determinants and military veterans' suicide ideation and attempt: A cross-sectional analysis of electronic health record data. J Gen Intern Med. 2020;35(6):1759-1767. The adverse social determinant variables were combined through principal component analysis to address multicollinearity.

**Table S2. Patient Characteristics in the Pre-Implementation Period in SCOUTT and non-SCOUTT Facilities (Fiscal Years 2016-2018)**

| **Characteristic** | **Overall**  **N = 31,354** | **SCOUTT**  **N = 3641** | **Non-SCOUTT**  **N = 27,713** | **p-value^1^** |
| --- | --- | --- | --- | --- |
| Age in years |  |  |  | 0.001 |
| 18-34 | 6636 (21.2%) | 702 (19.3%) | 5934 (21.4%) |  |
| 35-49 | 6500 (20.7%) | 715 (19.6%) | 5785 (20.9%) |  |
| 50-64 | 14,968 (47.7%) | 1826 (50.2%) | 13,142 (47.4%) |  |
| 65+ | 3250 (10.4%) | 398 (10.9%) | 2852 (10.3%) |  |
| Male sex | 28,966 (92.4%) | 3353 (92.1%) | 25,613 (92.4%) | 0.48 |
| Race and ethnicity |  |  |  | <0.001 |
| Non-Hispanic White | 19,292 (61.5%) | 2174 (59.7%) | 17,118 (61.8%) |  |
| Non-Hispanic Black | 8709 (27.8%) | 1103 (30.3%) | 7606 (27.4%) |  |
| Hispanic | 1720 (5.5%) | 207 (5.7%) | 1513 (5.5%) |  |
| Other | 1633 (5.2%) | 157 (4.3%) | 1476 (5.3%) |  |
| Marital status |  |  |  | 0.004 |
| Married | 5754 (18.4%) | 602 (16.5%) | 5152 (18.6%) |  |
| Single | 8826 (28.1%) | 1069 (29.4%) | 7757 (28.0%) |  |
| Previously married | 15,752 (50.2%) | 1868 (51.3%) | 13,884 (50.1%) |  |
| Missing | 1022 (3.3%) | 102 (2.8%) | 920 (3.3%) |  |
| Rural | 6233 (19.9%) | 463 (12.7%) | 5770 (20.8%) | <0.001 |
| VA priority group |  |  |  | 0.25 |
| Service-connected disability | 15,661 (49.9%) | 1851 (50.8%) | 13,810 (49.8%) |  |
| Very low income | 13,581 (43.3%) | 1556 (42.7%) | 12,025 (43.4%) |  |
| No service connection, copay required | 1540 (4.9%) | 181 (5.0%) | 1359 (4.9%) |  |
| Other | 572 (1.8%) | 53 (1.5%) | 519 (1.9%) |  |
| Literal homelessness^2^ | 25,284 (80.6%) | 2877 (79.0%) | 22,407 (80.9%) | 0.008 |
| Adverse SDOH^3^, median (IQR) | 5.0 (2.0-5.0) | 5.0 (2.0-5.0) | 5.0 (2.0-5.0) | <0.001 |
| Prior year NOSOS score^4^ |  |  |  | 0.009 |
| High | 4166 (13.3%) | 510 (14.0%) | 3656 (13.2%) |  |
| Medium | 17,612 (56.2%) | 2106 (57.8%) | 15,506 (56.0%) |  |
| Low | 7905 (25.2%) | 840 (23.1%) | 7065 (25.5%) |  |
| Missing | 1671 (5.3%) | 185 (5.1%) | 1486 (5.4%) |  |
| Medical conditions, median (IQR) | 2.0 (1.0-3.0) | 2.0 (1.0-3.0) | 2.0 (1.0-3.0) | 0.002 |
| Pain condition diagnosis | 19,472 (62.1%) | 2259 (62.0%) | 17,213 (62.1%) | 0.94 |
| Mental health and substance use disorders |  |  |  |  |
| Drug use disorder | 22,294 (71.1%) | 2587 (71.1%) | 19,707 (71.1%) | 0.94 |
| Alcohol use disorder | 18,810 (60.0%) | 2137 (58.7%) | 16,673 (60.2%) | 0.09 |
| Depression | 18,981 (60.5%) | 2263 (62.2%) | 16,718 (60.3%) | 0.03 |
| Anxiety | 11,137 (35.5%) | 1208 (33.2%) | 9929 (35.8%) | 0.002 |
| PTSD | 13,924 (44.4%) | 1752 (48.1%) | 12,172 (43.9%) | <0.001 |
| Serious mental illness | 7051 (22.5%) | 860 (23.6%) | 6191 (22.3%) | 0.08 |
| Other | 7918 (25.3%) | 866 (23.8%) | 7052 (25.4%) | 0.03 |

Abbreviations: IQR= Interquartile range; PTSD=Post-traumatic stress disorder; SDOH=Social determinants of health; SCOUTT=Stepped Care for Opioid Use Disorder Train the Trainer Initiative; VA=Veterans Affairs

Notes:

^1^p-values obtained from Pearson’s Chi-square test and Wilcoxon rank sum tests for categorical and numeric variables, respectively.

^2^Met study criteria for literal homelessness, compared to unstable housing. Evidence of literal homelessness includes an International Classification of Disease (ICD) version 10 diagnosis of homelessness; negative response to the question “In the past two months, have you been living in stable housing that you own, rent, or stay in as part of a household?”; residence in VA transitional housing; or receipt of community outreach, employment assistance or healthcare designated for homeless Veterans. Evidence of unstable housing includes ICD-10 diagnosis of inadequate housing or unstable housing among the housed; living in subsidized housing for formerly homeless veterans; or an affirmative response to the question “Are you worried or concerned that in the next two months you may not have stable housing that you own, rent, or stay in as part of a household?” Administrative data definitions provided in Table S1.

^3^Documented by ICD codes, structured text in clinical notes, or receipt of VA programs or services relating to (a) legal involvement; (b) exposure to violence; (c) military sexual trauma; (d) financial difficulties; (e) access to care concerns; (f) lack of social support; and (g) other unspecified psychosocial needs. Administrative data definitions provided in Table S1.

^4^NOSOS is a VA-derived risk adjustment score that predicts VA health care costs based on diagnosis-based hierarchical condition categories, pharmacy data, and demographic characteristics. Scores are centered so that 1.0 reflects the costs of a typical VA patient. We categorized log-transformed NOSOS scores as low (<0.0), medium (0.0-2.0) and high (≥2.0).

**Table S3. Homeless Experience Indicators, by Time (Pre-Implementation versus Post-Implementation) and Type of Facility (SCOUTT versus Non-SCOUTT)**

|  | **Pre-Implementation**  **Patients, n (%)** | | |  | **Post-Implementation**  **Patients, n (%)** | | |  |
| --- | --- | --- | --- | --- | --- | --- | --- | --- |
| **Homeless Experience Indicator** | **Overall N = 31,354** | **SCOUTT N = 3641** | **Non-SCOUTT N = 27,713** | ***p*** | **Overall N = 20,746** | **SCOUTT N = 2464** | **Non-SCOUTT N = 18,282** | ***p*** |
| Homeless diagnosis | 20,565 (65.6%) | 2439 (67.0%) | 18,126 (65.4%) | 0.06 | 13,180 (63.5%) | 1519 (61.6%) | 11,661 (63.8%) | 0.04 |
| Homeless screener response | 2216 (7.1%) | 253 (6.9%) | 1963 (7.1%) | 0.77 | 1092 (5.3%) | 119 (4.8%) | 973 (5.3%) | 0.30 |
| Health Care for Homeless Veteran program | 17,242 (55.0%) | 1865 (51.2%) | 15,377 (55.5%) | <0.001 | 10,148 (48.9%) | 1164 (47.2%) | 8984 (49.1%) | 0.08 |
| Community outreach | 43 (0.1%) | 1 (0.0%) | 42 (0.2%) | 0.06 | - | - | - | - |
| Grant & per diem | 6040 (19.3%) | 749 (20.6%) | 5291 (19.1%) | 0.03 | 3275 (15.8%) | 358 (14.5%) | 2917 (16.0%) | 0.07 |
| Transitional residence programs | 2602 (8.3%) | 232 (6.4%) | 2370 (8.6%) | <0.001 | 1577 (7.6%) | 133 (5.4%) | 1444 (7.9%) | <0.001 |
| Employment assistance | 1518 (4.8%) | 120 (3.3%) | 1398 (5.0%) | <0.001 | 880 (4.2%) | 47 (1.9%) | 833 (4.6%) | <0.001 |
| Unstable housing diagnosis | 19,597 (62.5%) | 2287 (62.8%) | 17,310 (62.5%) | 0.68 | 13,077 (63.0%) | 1590 (64.5%) | 11,487 (62.8%) | 0.10 |
| Unstable housing screener response | 1290 (4.1%) | 161 (4.4%) | 1129 (4.1%) | 0.32 | 660 (3.2%) | 74 (3.0%) | 586 (3.2%) | 0.59 |
| Permanent supportive housing | 13,166 (42.0%) | 1493 (41.0%) | 11,673 (42.1%) | 0.20 | 9077 (43.8%) | 1024 (41.6%) | 8053 (44.0%) | 0.02 |

Abbreviations: SCOUTT=Stepped Care for Opioid Use Disorder Train the Trainer Initiative

Note: P-values obtained from Pearson’s Chi-square test.

**Table S4. Social Determinant of Health Indicators, by Time (Pre-Implementation versus Post-Implementation) and Type of Facility (SCOUTT versus non-SCOUTT)**

|  | **Pre-Implementation**  **Patients, n (%)** | | |  | **Post-Implementation**  **Patients, n (%)** | | |  |
| --- | --- | --- | --- | --- | --- | --- | --- | --- |
| **Social Determinant Indicator** | **Overall N = 31,354** | **SCOUTT N = 3641** | **Non-SCOUTT N = 27,713** | **p** | **Overall N = 20,746** | **SCOUTT N = 2464** | **Non-SCOUTT N = 18,282** | **p** |
| History of legal involvement | 22,968 (73.3%) | 2684 (73.7%) | 20,284 (73.2%) | 0.50 | 14,637 (70.6%) | 1709 (69.4%) | 12,928 (70.7%) | 0.17 |
| Exposure to violence | 21,245 (67.8%) | 2505 (68.8%) | 18,740 (67.6%) | 0.15 | 13,345 (64.3%) | 1562 (63.4%) | 11,783 (64.5%) | 0.30 |
| Military sexual trauma | 3749 (12.0%) | 493 (13.5%) | 3256 (11.7%) | 0.02 | 3041 (14.7%) | 399 (16.2%) | 2642 (14.5%) | 0.002 |
| Financial difficulties | 24,539 (78.3%) | 2912 (80.0%) | 21,627 (78.0%) | 0.008 | 15,782 (76.1%) | 1888 (76.6%) | 13,894 (76.0%) | 0.49 |
| Access barriers | 2141 (6.8%) | 351 (9.6%) | 1790 (6.5%) | <0.001 | 1294 (6.2%) | 245 (9.9%) | 1049 (5.7%) | <0.001 |
| Lack of social support | 22,197 (70.8%) | 2595 (71.3%) | 19,602 (70.7%) | 0.50 | 14,152 (68.2%) | 1669 (67.7%) | 12,483 (68.3%) | 0.59 |
| Other psychosocial needs | 23,012 (73.4%) | 2695 (74.0%) | 20,317 (73.3%) | 0.36 | 14,594 (70.3%) | 1749 (71.0%) | 12,845 (70.3%) | 0.46 |

Abbreviations: SCOUTT=Stepped Care for Opioid Use Disorder Train the Trainer Initiative

Note: P-values obtained from Pearson’s Chi-square test.

**Figure S1-A. Covariate Balance between SCOUTT and Non-SCOUTT Groups before and after Weighting for the Outcome of Receiving MOUD Treatment**

Abbreviations: SCOUTT=Stepped Care for Opioid Use Disorder Train the Trainer Initiative; MOUD= Medication therapy for opioid use disorder; SDOH=Social determinants of health; HPACT=Homeless Patient Aligned Care Teams; OUD=Opioid use disorder
Notes: The figure depicts the standardized mean difference in covariates for patients who received care in a facility that participated in SCOUTT, compared to facilities that did not participate in SCOUTT. The red circles reflect the observed standardized mean difference, and the blue triangle reflects the difference after statistical weighting that attempts to render the two groups more comparable. The dashed lines at -0.1 and 0.1 serve as thresholds indicating an acceptable level of covariate imbalance.
The NOSOS is a risk-adjustment score, based on the Centers for Medicare and Medicaid Services (CMS) Hierarchical Condition Categories (HCC) risk adjustment model. The model includes hierarchical condition category codes, pharmacy data, and demographic characteristics. Scores are centered so that 1.0 reflects the costs of a typical VA patient. We categorized log-transformed NOSOS scores as low (<0.0), medium (0.0-2.0) and high (≥2.0).
The complexity score is a classification of VA facilities based on patient characteristics and the breadth of clinical and educational programs offered at the facility. The highest complexity facilities are those with highest patient volume, highest risk patients, large number of complex clinical programs, and large research and teaching programs. Medium-high complexity facilities have medium-high volume, medium and high risk patients, some complex clinical programs, and medium to large research and teaching programs; Medium complexity facilities have medium patient volume, low risk patients, few complex clinical programs, and no research or teaching programs; Low complexity facilities have low patient volume, low risk patients, few or no complex clinical programs, and no research or teaching programs.

**Figure S1-B. Covariate Balance between SCOUTT and Non-SCOUTT Groups before and after Weighting for the Outcome of Receiving Buprenorphine Treatment**

Abbreviations: SCOUTT=Stepped Care for Opioid Use Disorder Train the Trainer Initiative; MOUD= Medication therapy for opioid use disorder; SDOH=Social determinants of health; HPACT=Homeless Patient Aligned Care Teams; OUD=Opioid use disorder

Notes: The figure depicts the standardized mean difference in covariates for patients who received care in a facility that participated in SCOUTT, compared to facilities that did not participate in SCOUTT. The red circles reflect the observed standardized mean difference, and the blue triangle reflects the difference after statistical weighting that attempts to render the two groups more comparable. The dashed lines at -0.1 and 0.1 serve as thresholds indicating an acceptable level of covariate imbalance.

The NOSOS is a risk-adjustment score, based on the Centers for Medicare and Medicaid Services (CMS) Hierarchical Condition Categories (HCC) risk adjustment model. The model includes hierarchical condition category codes, pharmacy data, and demographic characteristics. Scores are centered so that 1.0 reflects the costs of a typical VA patient. We categorized log-transformed NOSOS scores as low (<0.0), medium (0.0-2.0) and high (≥2.0).

The complexity score is a classification of VA facilities based on patient characteristics and the breadth of clinical and educational programs offered at the facility. The highest complexity facilities are those with highest patient volume, highest risk patients, large number of complex clinical programs, and large research and teaching programs. Medium-high complexity facilities have medium-high volume, medium and high risk patients, some complex clinical programs, and medium to large research and teaching programs; Medium complexity facilities have medium patient volume, low risk patients, few complex clinical programs, and no research or teaching programs; Low complexity facilities have low patient volume, low risk patients, few or no complex clinical programs, and no research or teaching programs.

**Figure S1-C. Covariate Balance between SCOUTT and Non-SCOUTT Groups before and after Weighting for the Outcome of Receiving Methadone Treatment**

Abbreviations: SCOUTT=Stepped Care for Opioid Use Disorder Train the Trainer Initiative; MOUD= Medication therapy for opioid use disorder; SDOH=Social determinants of health; HPACT=Homeless Patient Aligned Care Teams; OUD=Opioid use disorder

Notes: The figure depicts the standardized mean difference in covariates for patients who received care in a facility that participated in SCOUTT, compared to facilities that did not participate in SCOUTT. The red circles reflect the observed standardized mean difference, and the blue triangle reflects the difference after statistical weighting that attempts to render the two groups more comparable. The dashed lines at -0.1 and 0.1 serve as thresholds indicating an acceptable level of covariate imbalance.

The NOSOS is a risk-adjustment score, based on the Centers for Medicare and Medicaid Services (CMS) Hierarchical Condition Categories (HCC) risk adjustment model. The model includes hierarchical condition category codes, pharmacy data, and demographic characteristics. Scores are centered so that 1.0 reflects the costs of a typical VA patient. We categorized log-transformed NOSOS scores as low (<0.0), medium (0.0-2.0) and high (≥2.0).

The complexity score is a classification of VA facilities based on patient characteristics and the breadth of clinical and educational programs offered at the facility. The highest complexity facilities are those with highest patient volume, highest risk patients, large number of complex clinical programs, and large research and teaching programs. Medium-high complexity facilities have medium-high volume, medium and high risk patients, some complex clinical programs, and medium to large research and teaching programs; Medium complexity facilities have medium patient volume, low risk patients, few complex clinical programs, and no research or teaching programs; Low complexity facilities have low patient volume, low risk patients, few or no complex clinical programs, and no research or teaching programs.

**Figure S1-D. Covariate Balance between SCOUTT and Non-SCOUTT Groups before and after Weighting for the Outcome of Receiving Naltrexone Treatment**

Abbreviations: SCOUTT=Stepped Care for Opioid Use Disorder Train the Trainer Initiative; MOUD= Medication therapy for opioid use disorder; SDOH=Social determinants of health; HPACT=Homeless Patient Aligned Care Teams; OUD=Opioid use disorder

Notes: The figure depicts the standardized mean difference in covariates for patients who received care in a facility that participated in SCOUTT, compared to facilities that did not participate in SCOUTT. The red circles reflect the observed standardized mean difference, and the blue triangle reflects the difference after statistical weighting that attempts to render the two groups more comparable. The dashed lines at -0.1 and 0.1 serve as thresholds indicating an acceptable level of covariate imbalance.

The NOSOS is a risk-adjustment score, based on the Centers for Medicare and Medicaid Services (CMS) Hierarchical Condition Categories (HCC) risk adjustment model. The model includes hierarchical condition category codes, pharmacy data, and demographic characteristics. Scores are centered so that 1.0 reflects the costs of a typical VA patient. We categorized log-transformed NOSOS scores as low (<0.0), medium (0.0-2.0) and high (≥2.0).

The complexity score is a classification of VA facilities based on patient characteristics and the breadth of clinical and educational programs offered at the facility. The highest complexity facilities are those with highest patient volume, highest risk patients, large number of complex clinical programs, and large research and teaching programs. Medium-high complexity facilities have medium-high volume, medium and high risk patients, some complex clinical programs, and medium to large research and teaching programs; Medium complexity facilities have medium patient volume, low risk patients, few complex clinical programs, and no research or teaching programs; Low complexity facilities have low patient volume, low risk patients, few or no complex clinical programs, and no research or teaching programs.

**Figure S2. Estimated SCOUTT Effect on the Probability of Receiving Medication Treatment for Opioid Use Disorder: Primary Mixed Effects and Facility Fixed Effect Sensitivity Analysis**

Abbreviations: SCOUTT=Stepped Care for Opioid Use Disorder Train the Trainer Initiative; ATT=Average treatment effect on the treated; EB=Entropy-balancing; MOUD=Medication therapy for opioid use disorder

Notes:

Points represent ATT estimates, and vertical bars indicate 95% confidence intervals. Estimates are expressed as differences in the probability of MOUD receipt, calculated for primary care patients with experience of homelessness (PEH) who received care in SCOUTT facilities compared with the counterfactual scenario in which the same facilities had not participated in SCOUTT. The horizontal dashed line at 0 denotes the null ATT.

Solid lines and dashed lines represent two estimating approaches:

(1) The solid line reflects the primary mixed effects approach. ATTs were estimated using quasi-Poisson regression models with entropy balancing weights applied to adjust for patient- and facility-level covariates, with random effects for patients and facilities.

(2) The dashed line reflects the facility fixed effect sensitivity analysis. ATTs were estimated using the same entropy balancing weights, with facility fixed effects replacing facility random effects. Standard errors were clustered at the patient level, and facility-specific predicted rates were aggregated using inverse standard error of the log-scale facility-specific estimates.

**Figure S3. Trends in Outcomes before and after SCOUTT Implementation Using Synthetic DiD**

Abbreviations: SCOUTT=Stepped Care for Opioid Use Disorder Train the Trainer Initiative; DiD=Difference-in-differences; MOUD= Medication therapy for opioid use disorder

Note: Each figure shows, on the y-axis, the average percentage of patients in the facility who obtained the applicable form of opioid use disorder treatment. The thinner solid lines represent the observed average facility-level treatment rates from 2016-2020, where the period from FY2016-2018 is before SCOUTT was implemented (i.e., “pre”) and FY2019-2020 covers the period after implementation (“post”). The thicker solid lines (blue and red) are designed to profile differences in MOUD treatment trends before and after implementation of the SCOUTT intervention. Dashed lines represent counterfactual trends, if SCOUTT had not been implemented. The difference between the observed and counterfactual values, represented with an arrow, is the DiD effect estimate.

The shaded area represents time weights in the pre-implementation period (FY2016–2018), used for constructing the synthetic control.

**Figure S4. Estimated SCOUTT Effect on the Probability of Receiving Medication Treatment for Opioid Use Disorder: Primary and Sensitivity Analysis of Synthetic DiD**

Abbreviations: SCOUTT=Stepped Care for Opioid Use Disorder Train the Trainer Initiative; DiD=Difference-in-differences; ATT=Average treatment effect on the treated; MOUD=Medication therapy for opioid use disorder

Notes:

Points represent ATT estimates, and vertical bars indicate 95% confidence intervals. Estimates are expressed as differences in the probability of MOUD receipt, calculated for primary care patients with experience of homelessness (PEH) who received care in SCOUTT facilities compared with the counterfactual scenario in which the same facilities had not participated in SCOUTT.

Solid lines and dot-dashed lines represent two estimating approaches:

(1) The solid line reflects the primary synthetic DiD analysis. This represents the difference in the percentage of PEH in a facility who received MOUD, calculated as the percent among PEH in SCOUTT facilities minus the expected percent who would receive MOUD under the counterfactual scenario, in the post-implementation years (FY2019-2020) minus the pre-implementation years (FY2016-2018). 95% confidence intervals were estimated via bootstrapping with 500 replications.

(2) The dot-dashed line reflects the sensitivity synthetic DiD analysis, where 4 of 30 SCOUTT facilities (13.3%) and 95 of 811 non-SCOUTT facilities (11.7%) with extreme pre-period slopes or small sizes were excluded to assess the regression-to-the-mean bias.

**Figure S5. Synthetic DiD Trends After Excluding Facilities with Extreme Pre-Implementation Slopes and Small Samples**

Abbreviations: SCOUTT=Stepped Care for Opioid Use Disorder Train the Trainer Initiative; DiD=Difference-in-differences; MOUD= Medication therapy for opioid use disorder

Note: The sensitivity analysis excludes 4 of 30 SCOUTT facilities (13.3%) and 95 of 811 non-SCOUTT facilities (11.7%) with extreme pre-period slopes or small sizes. Each figure shows, on the y-axis, the average percentage of patients in the facility who obtained the applicable form of opioid use disorder treatment. The thinner solid lines represent the observed average facility-level treatment rates from 2016-2020, where the period from FY2016-2018 is before SCOUTT was implemented (i.e., “pre”) and FY2019-2020 covers the period after implementation (“post”). The thicker solid lines (blue and red) are designed to profile differences in MOUD treatment trends before and after implementation of the SCOUTT intervention. Dashed lines represent counterfactual trends, if SCOUTT had not been implemented. The difference between the observed and counterfactual values, represented with an arrow, is the DiD effect estimate.

The shaded area represents time weights in the pre-implementation period (FY2016–2018), used for constructing the synthetic control.
